# Determination of Carotenoids and Their Antioxidant Activity in Fruits of Selected Species from the Genus *Rubus* and Their Cultivars and Hybrids from Poland Versus Other Regions of the World

**DOI:** 10.3390/antiox14121438

**Published:** 2025-11-28

**Authors:** Natalia Adamczuk, Mirosława Krauze-Baranowska, Marta Milewska, Katarzyna Kimel, Piotr Migas

**Affiliations:** Department of Pharmacognosy with Medicinal Plant Garden, Faculty of Pharmacy, Medical University of Gdańsk, Al. Gen. J. Hallera 107, 80-416 Gdańsk, Poland; natalia.adamczuk@gumed.edu.pl (N.A.);

**Keywords:** *Rubus*, species, cultivars, hybrids, fruits, carotenoids, antioxidant activity

## Abstract

The subject of the study were carotenoids, especially xantophylls in the fruits of various species/cultivars of raspberries, including those cultivated in Poland and those originating from other regions of the world (China, Finland): *Rubus occidentalis* (5 cultivars); *Rubus idaeus* (2 cultivars red fruited and 3 cultivars yellow fruited); *Rubus chamaemorus*; and *Rubus chingii* and hybrids: *R. occidentalis*/*R. idaeus* (2) and *R. idaeus*/*R. occidentalis* (1). Based on spectrophotometric analysis, the highest carotenoid content was found in cloudberry (*Rubus chamaemorus*) fruits, while the lowest was recorded for black raspberry cultivars. Similar carotenoid content results were obtained using thin-layer chromatography (TLC). The xantophyll profiles in the plant material studied were characterized—depending on the species/cultivar or hybrid—by the presence of β-apo-10′-luteinal, *trans*-lutein, and zeaxanthin. In addition, the antioxidant activity of the obtained hexane–acetone extracts were evaluated using DPPH, ABTS, and FRAP assays, as well as using the TLC-DB with DPPH radical. bioautography test.

## 1. Introduction

Raspberry fruits are eagerly consumed particularly due to their organoleptic properties—taste and aroma. It is believed that the positive effect of raspberries on human health is due to the presence of a complex of antioxidant compounds, especially anthocyanins, ellagitannins, flavonols and phenolic acids [1,2]. Most phytochemical studies on raspberries focus on polyphenolic compounds [3]. Data on the presence of carotenoids in raspberries and their role in antioxidant activity of these fruits are limited [4].

Many diseases of the 21st century are the result of oxidative stress, so antioxidants may play a key role in preventing their development. It is desirable to maintain a balance between antioxidants and reactive oxygen species (ROS), which are potentially harmful. Disruption of this balance leads to disorders and diseases such as cancer, diabetes, cardiovascular diseases, and accelerated aging [5].

The antioxidant capacity of carotenoids is determined by their chemical structure, specifically the presence of numerous conjugated double bonds [6,7]. The antioxidant action of carotenoids involves the deactivation of singlet oxygen (^1^O_2_) and scavenging of free radicals—agents responsible for cellular damage [8,9]. Carotenoids quench singlet oxygen either physically (by converting excess energy and dissipating it as heat through their lowest triplet state) or chemically (via a direct chemical reaction with the ^1^O_2_ molecule) [10]. Moreover, compounds with an unsubstituted β-ionone ring, like β-carotene can exhibit provitamin A activity [11]. A deficiency in vitamin A can lead to blindness and immune system dysfunctions [12]. Furthermore, two carotenoids, lutein and zeaxanthin, located in the retina, are responsible for absorbing high-energy blue light, thus protecting the retina from photodamage [13]. As antioxidants, they scavenge singlet oxygen and reactive oxygen species, helping to protect the structure of photoreceptor cell membranes from lipid peroxidation. As a result, they contribute to maintaining normal macular visual function. Lutein and zeaxanthin reduce the risk of age-related macular degeneration (AMD) and cataracts [14].

From a chemical point of view, carotenoids are tetraterpenes (C_40_ compounds) composed of eight isoprene units. They can be divided into two main groups: carotenes and xanthophylls. Carotenes are hydrocarbons made up solely of carbon and hydrogen atoms; this group includes, among others, β-carotene, α-carotene, and lycopene [15]. Xanthophylls are oxidized derivatives that, in addition to carbon and hydrogen atoms, also contain oxygen (as part of hydroxyl, ketone, or epoxide groups). This group includes lutein, zeaxanthin, and β-cryptoxanthin [16].

Carotenoids feature a characteristic conjugated system of double bonds in their structure—this chromophore is responsible not only for their color but also for the instability of these compounds in the presence of light, oxygen, or high temperatures [17].

To date, lutein, zeaxanthin, β-cryptoxanthin, α-carotene, and β-carotene have been identified as constituents of carotenoid complexes in most of the fruits of the different *Rubus idaeus* cultivars (red and yellow). There were no significant differences in the carotenoid profiles between the yellow- and red-fruited varieties [18]. Moreover, Abdul and Majeed revealed the following carotenoids in red raspberry (*Rubus idaeus* L.) fruit: neoxanthin and violaxanthin in addition to lutein and β-carotene. 9-cis-β-carotene was identified by Bradis in red raspberries [19]. Significant changes were observed in the profile of carotenoid compounds during the ripening period of raspberry fruit, namely a decrease in β-carotene and an increase in α-carotene [20]. Additionally, an increase in α-carotene levels was positively correlated with the level of the apocarotenoid α-ionone, while a decrease in β-carotene levels was negatively correlated with the level of β-ionone. Both low-molecular-weight apocarotenoids are responsible for the specific aroma of ripe raspberry fruits. The dioxygenase family is believed to be the carotenoid-cleaving enzymes (CCDs) and is presumably responsible for the degradation of carotenoids to apocarotenoids during raspberry fruit ripening [20]. In ripe fruit, the carotenoid complex was dominated by lutein and its esters, with low levels of zeaxanthin [18]. Perkins-Veazie and Fernandez analyzed by HPLC carotenoid complex in four *Rubus idaeus* L. cultivars and showed numerous peaks in the non-polar region, which were identified as esters of α-carotene, β-carotene, and possibly α- and β-cryptoxanthin after saponification [20].

The carotenoid profile of cloudberries (*Rubus chamaemorus*) is complex—in addition to neoxanthin, violaxanthin, antheraxanthin, lutein, zeaxanthin and β-carotene, the fruits have been shown to contain xanthophylls esterified simple chain fatty acids [21,22].

Fruits of *Rubus chingii* was found to have a unique carotenoid profile different from other raspberry species. In Chinese raspberry fruits, two xanthophylls (zeaxanthin and lutein) and three apocarotenoids as products their degradation were identified (β-citraurin and its esters—β-citraurin laurate and β-citraurin myristate). It was determined that, during ripening, the levels of apocarotenoids and zeaxanthin increased, while lutein levels gradually declined [23,24]. Raspberry is one of the few fruits in which massive production of apocarotenoids is observed during the ripening process, as indicated by Beekwilder, who observed an increase in the concentration of α- and β-ionones during ripening.

So far, the carotenoid complexes in the fruit of *Rubus occidentalis* have not been recognized.

The aim of the study was to identify complexes of carotenoid compounds and determine their contribution to the antioxidant activity of *Rubus idaeus* fruits of the yellow-fruited cultivars ‘Jantar’, ‘Poranna Rosa’, ‘Promyk’ and hybrid R1981802, as well as the red-fruited cultivars ‘Husaria’, ‘Delniwa’, and hybrid R1616002. Additionally, the study included *Rubus occidentalis* cultivars ‘Jewel’, ‘Bristol’, ‘MacBlack’, ‘Heban’, and ‘Niwot’, along with their hybrids: *R. occidentalis*/*R. idaeus* (R1613411 and R1613412), *R. idaeus*/*R. occidentalis* (R1314701) and also two other species from the genus *Rubus*, namely *R. chingii*, and *R. chamaemorus*. Carotenoid compounds were identified using a developed HPLC-DAD-ESI-MS method. Furthermore, some carotenoid compounds, as components of the studied complexes, were identified using the *off-line* TLC/HPLC-DAD-ESI-MS method. The antioxidant potential of hexane–acetone extracts was determined using DPPH, FRAP, and ABTS assays, as well as TLC bioautography. The carotenoid content was measured spectrophotometrically and compared with quantitative analysis results obtained using the TLC method.

## 2. Materials and Methods

### 2.1. Plant Material

The plant material coprised of fresh fruits from four cultivars of *Rubus occidentalis* L. (Rosaceae) = ‘MacBlack’, ‘Jewel’, ‘Niwot’, and ‘Heban’—as well as four related hybrids: R1613411, R1613409, R1613412, and R1314701. In addition, fruits from six cultivars of *Rubus idaeus* L. (Rosaceae) were collected. Yellow-fruited cultivars included ‘Poranna Rosa’, ‘Promyk’, ‘Jantar’, and the hybrid R1981802, while red-fruited cultivars comprised ‘Delniwa’, ‘Husaria’, and one of their hybrids, R1616002. All fruits were harvested in 2020 from plants grown at the Niwa Berry Breeding Company (Brzezna, Poland). Fruits of the ‘Bristol’ cultivar were sourced in 2020 from two producers: “Czarna malina” Barbara Rusiecka-Górniak (Nałęczów, Poland) and BiGrim Grzegorz Maryniowski (Łaziska, Poland). Additionally, fresh fruits of *Rubus chamaemorus* L. (Rosaceae) were obtained both from a commercial supplier in Finland and from plants cultivated by Lubelskie Zioła (Sosnówka, Poland) in 2021. All examined fruits were frozen at −20 °C, freeze-dried, and subsequently ground into powder. Dried fruits of *Rubus chingii* L. (Rosaceae) were purchased from commercial vendors in China (Table 1) and stored at room temperature in moisture-free conditions.

### 2.2. Reagents

Organic solvents of analytical grade (ethyl acetate, n-heptane, chloroform, methanol, hexane, acetone) were purchased from Avantor Performance Materials Poland S.A. (Gliwice, Poland). Analytical-grade formic acid (89–91% purity) was purchased from Merck (Darmstadt, Germany). HPLC-grade acetonitrile (LC/MS) Lichrosolv was purchased from Sigma-Aldrich (Steinheim, Germany). Deionized water was obtained using Elix/Synergy system (Merck-Millipore, Billerica, MA, USA). Tert-butylhydroquinone (TBHQ) was purchased from Sigma-Aldrich (St. Louis, MO, USA). Zeaxanthin, *trans*-lutein, β-cyptoxantin and β-carotene standards were purchased from Extrasynthese (Genay, France). In addition, zeaxanthin and lutein were isolated from dietary supplements, namely: Gold Lutein (Olimp Labs, Dębica, Poland), Strong-Zeaxanthin (Forest Vitamin created by nature, Kraków, Poland).

### 2.3. Preparation of Samples

Samples were prepared from 0.5 g of powdered, plant material by extracting with 25 mL of hexane or a solvent mixture of hexane–acetone (1:1, *v*/*v*) with or without the addition of TBHQ (0.1 g/99.9 mL extracting mixture), using an ultrasonic bath for 15 min in conditions without access to light. The obtained extracts were centrifuged in a laboratory centrifuge at 3000 rpm for 5 min and then decanted. The samples were evaporated to dryness under a reduced pressure at room temperature, and the dry residue was dissolved in either 1 mL of ethyl acetate or in 1 mL of methanol. The resulting extracts were filtered through a 0.22 µm membrane filter. Until analysis, the samples were stored in dark glass vials in a freezer (temperature −7 ± 1 °C, relative humidity 60–65%).

### 2.4. Methods

#### 2.4.1. Analysis of Carotenoids Content by Spectrophotometric Method

Ethyl acetate solutions of hexane–acetone (1:1, *v*/*v*) extracts were used for the analysis. The absorbance of the analyzed samples was measured using a spectrophotometer (Shimadzu, Japan) at a wavelength of λ—450 nm. Ethyl acetate was used as the blank sample. The total carotenoid content was calculated according to the formula [26]:

$$Carotenoid\;content\;\left(\mu g/g\right)\equiv \frac{A\times V(mL)\times 10^{4}}{A_{1cm}^{1\%}\times P(g)}$$

where A—absorbance; V—total volume of the extract; P—sample mass; A_1_ cm^1^%—specific absorption coefficient (2500).

#### 2.4.2. Analysis by Spectrophotometric Method of Antioxidant Activity

The antioxidant activity of the ethyl acetate solutions obtained from hexane–acetone (1:1, *v*/*v*) extracts was evaluated using three assays: the DPPH radical-scavenging test (2,2-diphenyl-1-picrylhydrazyl), the ABTS assay employing the diammonium salt of 2,2′-azobis(3-ethylbenzothiazoline-6-sulfonate), and the ferric reducing antioxidant power method (FRAP). All procedures followed protocols described previously in the literature [25].

##### Stable DPPH• Radical Assay

Antioxidant capacity was assessed using a 0.04 mM methanolic solution of the stable DPPH• radical. A calibration curve was created using six methanolic dilutions of Trolox (6-hydroxy-2,5,7,8-tetramethylchroman-2-carboxylic acid) at concentrations of 0.02, 0.04, 0.05, 0.06, 0.08, and 0.1 mM. Prior to measurement, the freshly prepared DPPH solution was kept at 4 °C for 60 min. For each determination, 2.5 mL of the DPPH reagent was mixed with 350 μL of the fruit extract in quartz cuvettes. The mixtures were incubated for 30 min in the dark at room temperature, after which absorbance was recorded at 517 nm.

##### Ferric Reducing Antioxidant Power (FRAP) Assay

The reducing potential toward Fe (III) ions was measured using the FRAP reagent, which was prepared by mixing (10:1:1, *v*/*v*) a 300 mM acetate buffer (pH 3.6), a 10 mM TPTZ solution (2,4,6-tris(2-pyridyl)-1,3,5-triazine) in 40 mM HCl, and a 20 mM FeCl_3_·6H_2_O solution. The reagent mixture was preheated to 37 °C before analysis. A standard curve was generated from six aqueous Trolox solutions (0.02, 0.03, 0.06, 0.12, 0.36, 0.48 mM). For each measurement, 3 mL of the FRAP reagent was combined with 150 μL of the fruit extract in quartz cuvettes. After incubating the mixtures for 30 min in the dark at room temperature, absorbance was measured at 593 nm.

##### ABTS•+ Radical Cation Assay

For the ABTS test, the ABTS•+ radical cation was produced by incubating a mixture of 2 mL of a 7 mM ABTS solution and 0.35 mL of a 140 mM potassium persulfate solution at 4 °C in darkness for 15 h. Following incubation, the mixture was diluted with redistilled water (1:90, *v*/*v*) to obtain an absorbance of 0.7 ± 0.02. A calibration curve was prepared using six Trolox solutions in water at concentrations of 0.02, 0.03, 0.05, 0.08, 0.1, and 0.12 mM. For each analysis, 2 mL of the diluted ABTS reagent was mixed with 200 μL of the sample in quartz cuvettes. Absorbance was measured twice at 734 nm, 6 min after initiating the reaction.

#### 2.4.3. TLC Analyses

Separation of ethyl acetate solutions of hexane–acetone extracts (1:1, *v*/*v*) with the addition of 0.1% TBHQ was carried out on TLC Si60 chromatography plates (20 cm × 20 cm) (Merck, Darmstadt, Germany) trimmed with a TLC-cuter (OM Laboratory, Chigasaki, Japan) to 5 cm × 20 cm. The plates were activated by heating on a TLC plate heater (Camag, Muttenz, Switzerland) at 120 °C for 20 min. Analyzed samples were applied to the TLC plates as 8 mm bands, at 5 mm intervals using a semi-automatic DESAGA Sampler AS 30 autosampler (Sarstedt, Germany) under an inert gas-nitrogen atmosphere. Separations were carried out in a Twin Trough Chamber (Twin Trough Chamber 20 cm × 10 cm; Camag, Muttenz, Switzerland) at a constant temperature (4 °C ± 1 °C) and humidity of 60–65% and protected from light. For quantitative and qualitative analysis, the separation of carotenoids was carried out using a mixture of n-heptane: ethyl acetate: acetone (65:20:20, *v*/*v*) as mobile phase. Semipreparative TLC was performed on TLC Si60 F_254_ chromatography plates with a use of n-heptane: ethyl acetate: acetone (60:20:20, *v*/*v*) as mobile phase. Separations were carried out on a distance of 50 mm (without saturation of chromatographic chamber). After development of the chromatograms, the mobile phase was evaporated for 5 min at room temperature. The resulting chromatograms were analyzed and archived in daylight using a TLC Visualizer videoscanner (Camag, Muttenz, Switzerland) and the winCATS 1.4.9.2001 program.

##### TLC–DB with DPPH Radical

The TLC chromatograms obtained under the above-mentioned conditions were immersed in a DPPH solution using a Chromatogram immersion device III (Camag, Muttenz, Switzerland), according to the method described in the literature [27,28]. The developed chromatograms were examined in daylight 5 min and 18 h after immersion. Areas forming yellow bands on the purple background of the chromatographic plate were identified as antioxidants.

##### Quantitative TLC Analyses

Stock solutions of the standard compounds were obtained by dissolving 1 mg of β-carotene and β-cryptoxanthin in 1 mL of ethyl acetate each. For TLC analyses, dilutions of stock solutions of both standards in ethyl acetate corresponding to 0.008 mg/mL, 0.030 mg/mL and 0.120 mg/mL were prepared. 10 µL solutions of ethyl acetate solutions of hexane–acetone extracts were applied as 8 mm bands at 16 mm intervals (*n* − 3) alternating with bands of the standard substances β-carotene and β-cryptoxanthin at 150 ng, 300 ng and 600 ng per spot. The resulting chromatograms were analyzed using a TLC Visualizer videoscanner (Camag, Muttenz, Switzerland) under the control of the winCATS 1.4.9.2001 software. The content of carotenoids was calculated on β-cryptoxanthin.

The method was validated by establishing the limits of detection (LOD) and quantification (LOQ) for β-carotene and β-cryptoxanthin, as well as assessing precision and linearity, in accordance with the International Council for Harmonization (ICH) guideline Q2(R2) for analytical procedure validation (Table 2).

##### Linearity, Limit of Detection (LOD) and Limit of Quantification (LOQ)

Increasing amounts (40, 80, 150, 300, 600, 1200 ng/spot) of β-cryptoxanthin standard were applied in duplicates to a TLC plate with a silica gel layer. A correlation was plotted between the peak area of the standard and its applied amount. The detection limit was determined based on the visual analysis of the plates and considering that the ratio of the signal to the noise was S/N = 3:1. LOQ was the experimentally determined amount of β-carotene and β-cryptoxanthin standard in the band whose S/N = 10:1 (Table 2).

##### Precision

Precision was evaluated as the relative standard deviation (RSD) obtained from multiple independent measurements of β-cryptoxanthin in the extract of the *Rubus idaeus*/*Rubus occidentalis* hybrid R1314701, which exhibited the most complex carotenoid profile. Intra-day precision was assessed from three separate analyses of the extract, each applied to the same plate in triplicate (*n* = 9). Intermediate (inter-day) precision was determined from three independent measurements, also spotted in triplicate, performed on three consecutive days (*n* = 9). An RSD value of ≤20% was considered acceptable [29,30].

### 2.5. HPLC-DAD-ESI-MS Analysis

Methanolic solutions of hexane–acetone extracts of fruits from the tested cultivars and hybrid obtained by semi-preparative TLC and methanolic solutions of hexane–acetone extracts without TBHQ and were used for analysis. HPLC were analyzed using an LC system from Shimadzu (Kyoto, Japan) consisting of two LC-20AD pumps, a semi-micro mixer, a CBM20A system controller, a CT0-20AC column thermostat, a SIL 20ACXR autosampler, a SPD-M20A UV/vis (Diode Array Detector), and an LCMS-2020 mass spectrometer with ESI ionization. Data were collected and processed by LabSolution software (version 1.2). Analysis was performed on a Kinetex C-18 column (2.6 µm, 2.1 × 150 mm) (Phenomenex, Torrance, CA, USA) using gradient elution according to the program (% B in A): A: acetonitrile–water 50:50 (*v*/*v*) with 0.1% formic acid, B: acetonitrile with 0.1% formic acid:

- 0 min–50% B;
- 10 min–88% B (linear increase from 50 to 88%);
- 20 min–100% B (linear increase from 88 to 100%);
- 50 min–100% B hold at 100%;
- 52 min–50% B: linear decrease from 50 to 100%;
- 70 min–50% B: re-equilibrate

at a mobile phase flow rate of 0.2 mL/min, column temperature—35 °C, injection volume: 1 µL, UV detection at λ 450 nm.

Mass spectra were obtained in positive ion (PI) modes. Full scan (*m*/*z* 200–800 range) and SIM (selected ion monitoring) techniques were used to monitor specific signals. Operating parameters: interface voltage 5.0 kV, detector voltage 1.3 kV, thermal block 200 °C, DL temperature (desolvation line) 250 °C, nebulising gas flow (N2) 1.5 L/min, drying gas flow 10 L/min.

### 2.6. Off-Line TLC/HPLC-DAD-ESI-MS Analysis of Carotenoids from Selected Raspberry Fruits and Dietary Supplements

Ethyl acetate solutions of hexane–acetone extracts, from the fruits of *R. idaeus* ‘Jantar’ and *R. idaeus* ‘Poranna Rosa’ were used for the study. The ethyl acetate solutions were applied on a single plate (*n* − 3) in a volume of 420 µL each, along the longer edge of a TLC Si60 F_254_ plate (5 cm × 20 cm) as 10 mm bands, with a distance 5 mm between them. Chromatograms were developed using a mobile phase of n-heptane: ethyl acetate: acetone (65:20:20, *v*/*v*) over a distance of 50 mm. From the obtained TLC chromatograms from the fruits of the ‘Jantar’ cultivar, two yellow-colored bands were scraped off, namely: a band with an R_F_ value of 0.35—band I, and a band with an R_F_ value of 0.39—band IV. From the fruits of the ‘Poranna Rosa’ cultivar, a band with an R_F_ value of 0.39 was isolated (band IV). The carotenoid bands were scraped off the silica gel layer and extracted with 0.5 mL of ethyl acetate using a shaker for 4 min (1.600 rpm). The samples were centrifuged (10.000 rpm, 10 min), and the solvent was evaporated from the collected supernatant, under a stream of argon. The dry residue was re-dissolved in 200 µL of methanol and subjected to HPLC-DAD-ESI-MS analysis. Under the conditions described above, zeaxanthin and lutein were isolated from dietary supplements. Solutions of ethyl acetate extracts (hexane–acetone) from the dietary supplement Gold Lutein and Strong Zeaxanthin were analyzed using the chromatographic system described above. From the resulting chromatograms, yellow-colored bands visible in daylight were scraped off—specifically, from the Gold Lutein supplement, band I corresponding to the *trans*-lutein standard with an R_F_ 0.35; and from the Strong Zeaxanthin supplement, two bands—Band II with an (R_F_ 0.33), corresponding to the zeaxanthin standard and unknown Band III with an R_F_ value of 0.38.

### 2.7. Statistical Analysis

The average variations in carotenoid content and antioxidant activity were assessed using a one-way analysis of variance (ANOVA), followed by Tukey’s Post Hoc multiple comparison test. Statistical significance was defined as *p* < 0.05. All calculations were carried out with the Statistica 12 software package (StatSoft, Krakow, Poland).

## 3. Results

### 3.1. Determination of Carotenoids in Fruits of the Genus Rubus

#### Determination of Total Carotenoids Content

The highest carotenoid content was found in *R. chamaemorus* fruits, regardless of the origin of the raw material (Poland and Finland), ranging from 46.01 to 46.27 mg/100 g DW. A high carotenoid concentration was also observed in the hybrid *Rubus occidentalis*/*Rubus idaeus* R1613411—29.79 mg/100 g DW. The carotenoid content in the analyzed *R. idaeus* and *R. occidentalis* cultivars was similar, ranging from 7.36 mg/100 g DW (‘Delniwa’) to 20.14 mg/100 g DW (‘Jantar’) for *R. idaeus*, and from 7.24 mg/100 g DW (‘Bristol’) to 12.31 mg/100 g DW (‘Jewel’) for *R. occidentalis*, respectively. The carotenoid content in two hybrids, *R. idaeus*/*R. occidentalis* R1314701 (17.55 mg/100 g DW) and *R. occidentalis*/*R. idaeus* R1613412 (12.98 mg/100 g DW), was higher than in the black raspberry cultivars but remained comparable to that found in the hybrid *R. idaeus* R1616002 (8.69 mg/100 g DW). The lowest carotenoid concentration was recorded in *R. chingii* fruits—4.51 mg/100 g DW (Table 3).

### 3.2. Antioxidant Activity

#### 3.2.1. DPPH

Among the analyzed raspberry fruits from various cultivars and hybrids, the highest antioxidant activity determined using the DPPH method was observed in the carotenoid complexes from hexane–acetone (1:1 *v*/*v*) extracts of *Rubus chamaemorus* fruits (0.119–0.122 mmol TE/g DW), as well as in cultivated *R. idaeus* varieties, both yellow-fruited—‘Promyk’ (0.118 mmol TE/g DW), ‘Jantar’ (0.115 mmol TE/g DW)—and red-fruited—‘Husaria’ (0.122 mmol TE/g DW) and ‘Delniwa’ (0.115 mmol TE/g DW). The yellow-fruited cultivar ‘Poranna Rosa’ showed slightly lower antioxidant potential (0.095 mmol TE/g DW) compared to the other yellow varieties. Carotenoid complexes from *R. occidentalis* cultivars exhibited nearly half the antioxidant activity in the DPPH radical scavenging assay: ‘Heban’ and ‘Niwot’ (0.087 mmol TE/g DW), ‘MacBlack’ (0.072 mmol TE/g DW), ‘Bristol’ (0.066 mmol TE/g DW), and ‘Jewel’ (0.060 mmol TE/g DW). Among the tested hybrids, *R. occidentalis*/*R. idaeus* R1613412 (0.070 mmol TE/g DW) and *R. occidentalis*/*R. idaeus* R1613411 (0.063 mmol TE/g DW) showed antioxidant potential similar to that of black raspberry cultivars. An exception was the hybrid *R. idaeus*/*R. occidentalis* R1314701 (0.096 mmol TE/g DW), which exhibited antioxidant activity comparable to that of the *R. idaeus* cultivars.

#### 3.2.2. ABTS

The free radical scavenging potential was also assessed using the ABTS reagent. The results obtained for the individual varieties and cultivars in terms of ABTS radical scavenging activity were similar to those associated with DPPH radical scavenging, namely, the strongest antioxidant activity was exhibited by the carotenoid complexes from *R. idaeus* cultivars ‘Promyk’ (0.47 mmol TE/g DW), ‘Husaria’ (0.46 mmol TE/g DW), ‘Delniwa’ (0.41 mmol TE/g DW), and ‘Jantar’ (0.40 mmol TE/g DW), as well as from *R. chamaemorus* (0.41–0.43 mmol TE/g DW). Among the black raspberry varieties, ‘Heban’ stood out with relatively high activity (0.34 mmol TE/g DW). The carotenoid-rich fruits of the *R. idaeus* variety ‘Poranna Rosa’ (0.24 mmol TE/g DW) and black raspberry varieties had twice as low activity, ranging from 0.27 mmol TE/g DW (‘Niwot’, ‘Bristol’) to 0.2 mmol TE/g DW (‘Jewel’). Among the tested hybrids, as in the DPPH assay, the carotenoid complex from *R. idaeus*/*R. occidentalis* R1314701 displayed the highest antioxidant potential (0.41 mmol TE/g DW). The remaining hybrids showed nearly twofold lower activity, namely, 0.25 mmol TE/g DW (*R. occidentalis*/*R. idaeus* R1613412 and *R. idaeus* R1616002) and 0.17 mmol TE/g DW (*R. occidentalis*/*R. idaeus* R1613411).

#### 3.2.3. FRAP

The highest free radical inhibition activity was observed for the hexane–acetone extract from *R. idaeus* ‘Husaria’ fruits (0.8 mmol TE/g DW). Seven of the analyzed hexane–acetone extracts—from *R. idaeus* (‘Jantar’, ‘Promyk’), *R. chamaemorus* (2 samples), and hybrids of *R. occidentalis* and *R. idaeus* (R1613411, R1314701, R1616002)—showed similar antioxidant activity ranging from 0.45 to 0.55 mmol TE/g DW. Only two carotenoid sets among the tested *R. idaeus* cultivars, namely ‘Delniwa’ and ‘Poranna Rosa’, exhibited lower activity, at 0.33 mmol TE/g DW and 0.26 mmol TE/g DW, respectively. Fruits of *R. occidentalis* species, as observed in other tests, showed considerably weaker antioxidant activity, ranging from 0.2 to 0.32 mmol TE/g DW.

### 3.3. Analysis of Carotenoids in Dietary Supplements and Reference Substances Using the Off-Line TLC/HPLC Method

Analysis of carotenoids in dietary supplements was performed against standard compounds. Methanolic solutions of zeaxanthin (2), β-cryptoxanthin (9), and β-carotene (10) were analyzed using HPLC-DAD-ESI-MS and TLC in order to determine RF values, retention times (tR), UV absorption maxima, and molecular ion values in ESI-MS spectra. Under ESI (“electrospray”) ionization conditions, in addition to protonated molecular ions [M + H]^+^, radical ions [M]^+^·were observed in the mass spectra of carotenoids. Their presence in the ESI spectra of carotenoids is described in the available literature—xanthophylls easily form radical ions due to their long skeleton containing many conjugated unsaturated bonds and protonated molecular ions due to their oxygen-containing functional groups [19]. The collected data are presented below in Table 4.

**Figure 1:**
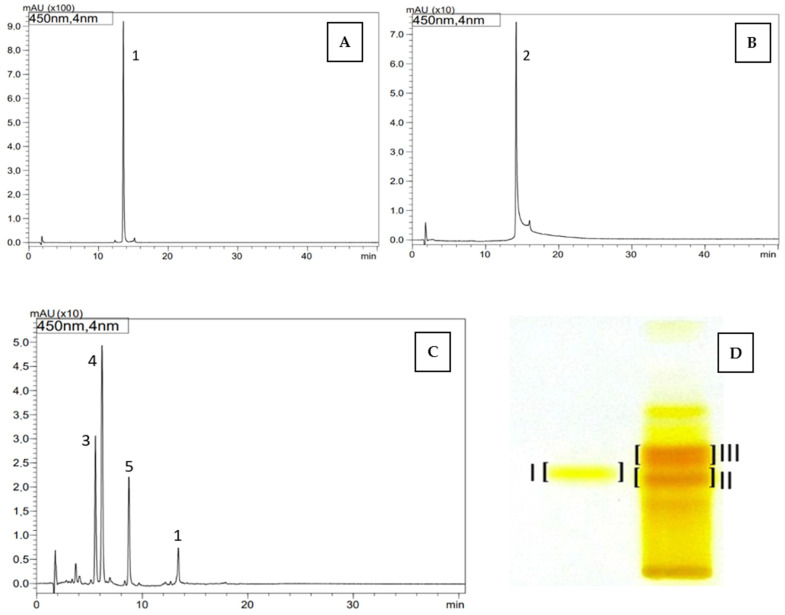
HPLC chromatograms (**A**–**C**) and TLC chromatogram (**D**) of carotenoids identified in dietary supplements namely, Gold Lutein (**A**,**D**, band I) and Strong Zeaxanthin (**B**–**D**, band II and III): 1—lutein, 2—zeaxanthin, 3—β-apo-12’-zeaxanthinal, 4—β-apo-10’-zeaxanthinal, 5—β-apo-8’-zeaxanthinal, TLC: band I—*trans* lutein (1), band II—zeaxanthin (2), band III—zeaxanthinals (3–5).

Carotenoid compounds present in dietary supplements were analyzed against standards using the *off-line* TLC/HPLC-DAD-ESI-MS method to confirm their identity. As a result, the presence of *trans*-lutein (**1**) (TLC R_F_—0.35; band I, HPLC t_R_—13.56 min) was confirmed in the dietary supplement Gold Lutein, and zeaxanthin (**2**) (R_F_—0.33; band II, HPLC t_R_—14.17 min) was confirmed in the supplement Strong Zeaxanthin. Additionally, the presence of a zeaxanthinal complex (TLC R_F_—0.38; band III) was demonstrated in the Strong Zeaxanthin supplement. Based on the obtained UV spectra and *m*/*z* values of protonated molecular ions [M + H]^+^, the compound with t_R_—5.56 min and *m*/*z* at 367 was identified as β-apo-12’-zeaxanthinal (**3**), the compound with t_R_—6.19 min and *m*/*z* at 393 as β-apo-10’-zeaxanthinal (**4**), and the compound with t_R_—8.74 min and *m*/z at 433 as β-apo-8’-zeaxanthinal (**5**) (Table 5, Figure 1).

### 3.4. Analysis of Carotenoids from Some Analyzed Cultivars and Hybrids of the Genus Rubus Using Off-Line TLC/HPLC-DAD-ESI-MS

Based on the results of the HPLC-DAD-ESI-MS analyses, in the prepared bands IV, obtained by the semi-preparative TLC method, from both analyzed cultivars—‘Poranna Rosa’, ‘Jantar’, the presence of a compound with a molecular weight of 392 Da was demonstrated, based on the presence of a protonated molecular ion [M + H]^+^ at *m*/*z* 393 in the ESI-MS spectrum. Furthermore, the compound had one absorption maximum in the UV spectrum at λ_max_ 424 nm. On this basis, the compound was identified as β-apo-10′-luteinal (6), which is consistent with the literature data [31]. In the prepared band I from the ‘Jantar’ cultivar, the presence of trans-lutein (1) as the dominant one was confirmed, accompanied by low concentrations of β-apo-10′-luteinal (6) and *cis*-lutein (7). In the UV spectra, both *trans*-lutein (1) and *cis*-lutein (7) showed absorption maxima in the ranges characteristic for lutein λ_max_ 260–266 and 325–330, 420–421 (sh), 443–444, 472–474 nm but they differed in their intensity, namely the trans isomer is characterized by a higher intensity of the maximum at 260–266 nm to the maximum at 325–330 nm compared to the cis isomer. In both bands I and IV, a carotenoid compound was present in the HPLC chromatograms with a retention time tR of 14.55 min and a UV spectrum characteristic for lutein derivatives (Table 4, Figure 1). Based on literature data and the fragment ion [M-caprylate]+ present in the ESI-MS spectrum, at *m*/*z* 677, the compound is probably an isomer of lutein dicaprylate [32]. Moreover, in band IV, alongside the dominant β-apo-10′-luteinal, small amounts of *cis*- and *trans*-lutein were also present. The results of HPLC-DAD-ESI-MS analyses clearly indicate that the carotenoid bands separated by TLC are mixtures, i.e., they contain a dominant compound accompanied by other carotenoids, most often present in lower concentrations.

### 3.5. HPLC-DAD-ESI -MS Analysis of Carotenoids from Some Analyzed Cultivars and Hybrids of the Genus Rubus

**Figure 2:**
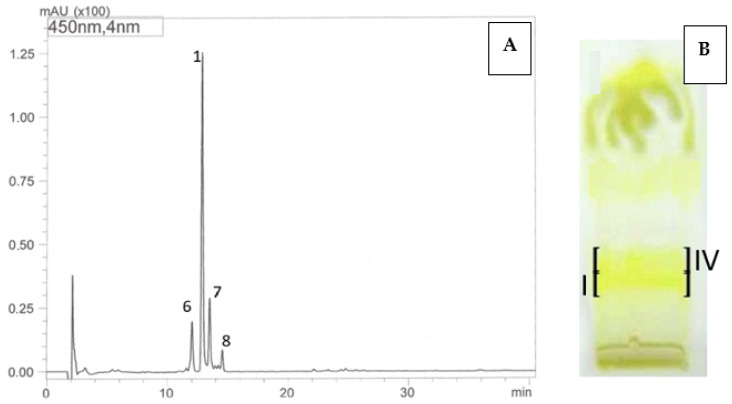

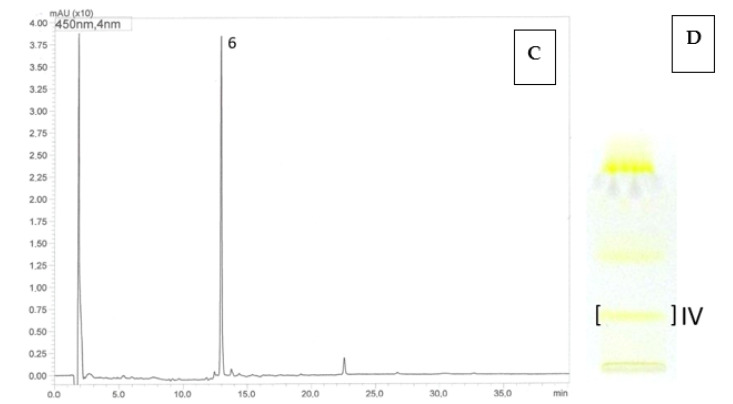
HPLC chromatograms (**A**,**C**) and TLC chromatogram (**B**,**D**). Identification of carotenoids in *R. idaeus* ‘Jantar’ (**A**,**B**) and *R. idaeus* ‘Poranna Rosa’ (**C**,**D**) using the off-line TLC/HPLC-DAD-ESI-MS method: 6—β-apo-10’-apoluteinal, 1—*trans*-lutein, 7—*cis*-lutein, 8—isomer of lutein dicaprylate, *R. idaeus* TLC ‘Jantar’: band I—*trans*-lutein (1), β-apo-10’-apoluteinal (6), *cis*-lutein (7), isomer of lutein dicaprylate (8), band IV: β-apo-10’-apoluteinal (6), *trans*-lutein (1), *cis*-lutein (7), isomer of lutein dicaprylate (8); *R. idaeus* TLC ‘Poranna Rosa’ band IV—β-apo-10’-apoluteinal (6).

**Figure 3:**
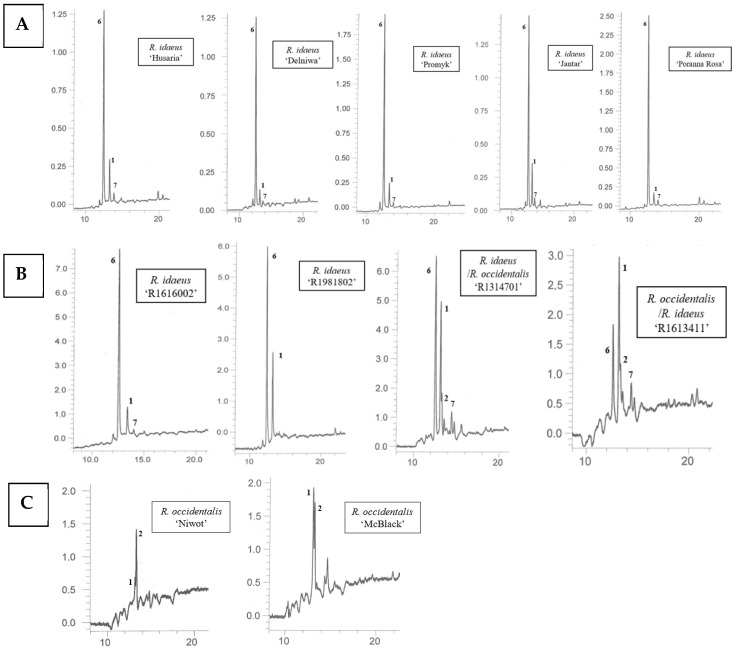
HPLC chromatograms of carotenoids in fruits of *R. idaeus* ‘Husaria’, ‘Delniwa’, ‘Promyk’, ‘Jantar’, ‘Poranna Rosa’ (**A**), hybrids: *R. idaeus* R1616002, *R. idaeus* R191802, *R. idaeus*/*R. occidentalis* R1314701, *R. occidentalis*/*R. idaeus* R1613411 (**B**), and *R. occidentalis:* ‘Niwot’, ‘MacBlack’ (**C**): 1—*trans*-lutein, 2—zeaxanthin, 6—β-apo-10’-apoluteinal, 7—*cis*-lutein.

Using the HPLC-DAD-ESI-MS method, carotenoids were identified in methanolic solutions of hexane–acetone extracts from fruits of 10 cultivars namely: *R. idaeus*, ‘Delniwa’, ‘Husaria’ (red fruits), ‘Jantar’, ‘Poranna Rosa’, ‘Promyk’ (yellow fruits), *R. occidentalis*: ‘Niwot’, ‘McBlack’ (black fruits) and hybrids *R. occidentalis*/*R. idaeus* R1613411; *R. idaeus*/*R. occidentalis* R1314701; *R. idaeus* R1616002 (red fruits), *R. idaeus* R1981802. Due to the low solubility of carotenoids in methanol, also limited by the presence of ballast substances (fatty oils) in the extracts, as well as their low concentrations in some fruits (Figure 2), these compounds could not be detected in the fruits of the following species, namely: *R. occidentalis* ‘Bristol’, ‘Heban’ and ‘Jewel’, *R. chamaemorus* from both collection sites (Finland and Poland), as well as in the fruits of *R. chingii* and the *R. occidentalis*/*R. idaeus* hybrid R1613412. As a result of performed HPLC analyses, β-apo-10′-luteinal (6) was found to be the dominant compound in all *Rubus idaeus* fruits, alongside *trans*-lutein (1) occurring in lower concentrations (Figure 3). The carotenoid profile of the *R. idaeus* hybrids R1616002 and *R. idaeus* R1981802 was identical to the carotenoid complex observed in red and yellow raspberries, although *trans*-lutein (1) was present in comparatively higher concentrations in the latter. In contrast, in the *R. idaeus*/*R. occidentalis* R1314701 and *R. occidentalis*/*R. idaeus* R1613411 hybrids, alongside β-apo-10′-luteinal (6) and lutein (1), zeaxanthin (2) was also present. Black raspberry fruits—‘Niwot’, ‘McBlack’ had a distinctly different carotenoid profile; zeaxanthin (2) and trans-lutein (1) were present in both cultivars, with zeaxanthin (2) dominating in the former and lutein (1) in the latter (Figure 3).

### 3.6. TLC Analysis of Carotenoids in Analyzed Fruits from Different Cultivars and Hybrids from the Genus Rubus

**Figure 4:**
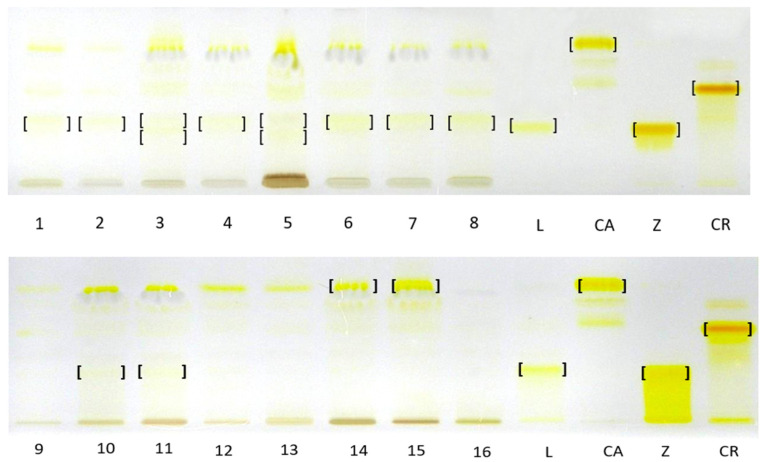
TLC chromatograms of carotenoid complexes in hexane–acetone extracts from fruits of different *Rubus* species and their cultivars/hybrids and carotenoid standards. L—*trans* lutein, CA—β-carotene, Z—zeaxanthin, CR—β-cryptoxanthin, 1—‘Husaria’, 2—‘Delniwa’, 3—R1314701, 4—R1616002, 5—R1613411, 6—‘Jantar’, 7—‘Promyk’, 8—‘Poranna Rosa’, 9—‘Bristol’, 10—‘Niwot’, 11—‘MacBlack’, 12—‘Jewel’, 13—R1613412, 14—*R. chamaemorus* Poland, 15—*R. chamaemorus* Filnland, 16—*R. chingii*. Stationary phase—TLC Silica Gel 60 glass plates; mobile phase—n-heptane:ethyl acetate:acetone 65:20:20, *v*/*v.*

TLC analysis against standard compounds, including isolated and identified β-apo-10′-luteinal (6) confirmed the presence of zeaxanthin (2) as the dominant xanthophyll complex in *R. occidentalis* ‘Niwot’ fruits and *trans*-lutein (1) in *R. occidentalis* ‘MacBlack’ fruits. Furthermore, it confirmed the presence of β-apo-10′-luteinal (6) as the dominant one in yellow raspberry fruits, namely the cultivars ‘Jantar’, ‘Poranna rosa’, ‘Promyk’, and red fruits of the cultivars ‘Husaria’, ‘Delniwa’, in addition to the fruits of hybrids *R. idaeus* R1616002 and R1981802. The compound band was also present in the hybrid *R. occidentalis*/*R. idaeus* R1613411; *R. idaeus*/*R. occidentalis* R1314701; next to the unresolved and co-eluting *trans*-lutein (1) and zexanthin (2) bands, which is consistent with the HPLC-DAD-ESI-MS results. Due to the very low concentrations of *trans*-lutein in the tested plant raw material, as demonstrated by the HPLC-DAD-ESI-MS method, the bands of this carotenoid were invisible in the TLC chromatograms in most raspberry cultivars. In relation to the β-carotene standard, the presence of this compound was confirmed only in the cloudberry fruit complex. In the remaining fruits, due to the lack of appropriate standards and the poor resolution of the developed TLC method, and sometimes low concentrations of carotenoids, it was not possible to identify the entire complex of carotenoid compounds present in them (Figure 4).

### 3.7. Determination of the Antioxidant Activity of Carotenoids in the Analyzed Raspberries by the TLC-DB

**Figure 5:**
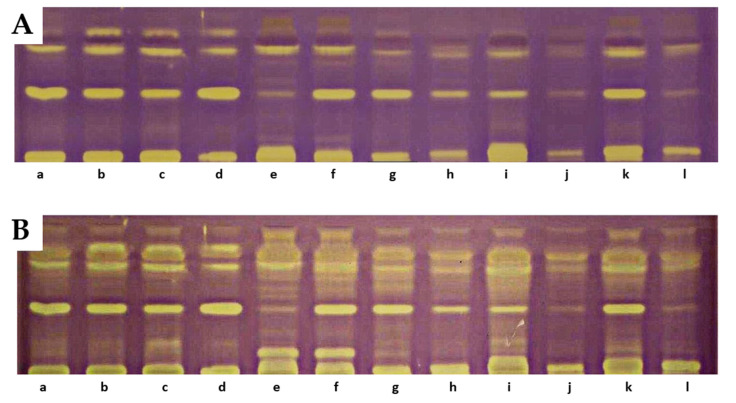

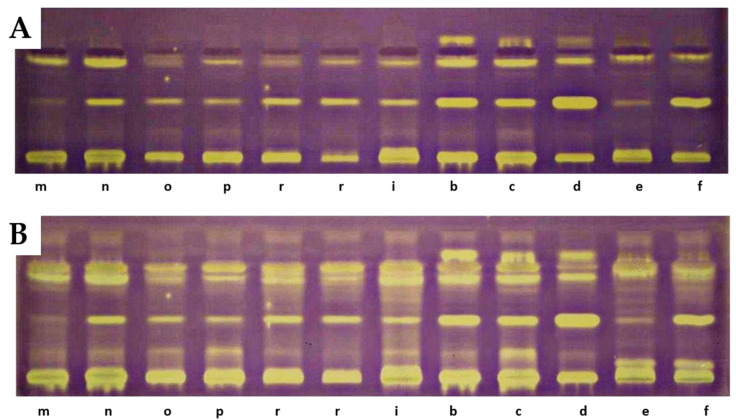
Results of TLC-DB bioautography of the tested extracts after 5 min (**A**) and 18 h (**B**). a—‘Delniwa’, b—‘Jantar’, c—‘Promyk’, d—‘Poranna Rosa’, e—*R. chamaemorus* Finland, f—*R. chamaemorus* Poland, g—*R. chingii*, h—‘MacBlack’, i—‘Bristol’, j—‘Jewel’, k—R1314701, l—R1613411, m—R1616002, n—‘Husaria’, o—R1613412, p—‘Heban’, r—‘Niwot’. Stationary phase—TLC Silica Gel 60 glass plates; mobile phase—n-heptane:ethyl acetate:acetone 65:20:20, *v*/*v.*

On the TLC chromatograms of extracts from the studied fruits treated with the DPPH radical, the presence of yellow-colored bands with antioxidant properties was observed, some of which remained at the starting line. At the positions corresponding to reference lutein, zeaxanthin, or β-apo-10′-luteinal, no yellow bands indicative of antioxidant activity were detected. A particularly intense band of an unidentified compound, present in all analyzed extracts at an R_F_ value of 0.42, was noted. The intensity of the bands increased after 18 h, with additional bands exhibiting DPPH radical-scavenging properties also appearing [33]. The greatest number of yellow-colored bands after 18 h was recorded for *R. chamaemorus*, *R. chingii*, *R. occidentalis* ‘Bristol’, R. idaeus ‘Jantar’, ‘Promyk’, ‘Poranna Rosa’, and the hybrid *R. idaeus × R. occidentalis* R1314701. The cultivars characterized by the lowest number of yellow-colored bands were *R. occidentalis* ‘MacBlack’ and ‘Jewel’, as well as the hybrids *R. idaeus* R1616002, *R. occidentalis × R. idaeus* R1613411, and *R. occidentalis × R. idaeus* R1613412. The TLC profiles of carotenoid compounds in the studied raspberries indicate stronger antioxidant activity of compounds with R_F_ values above 0.5 compared to carotenoid compounds with R_F_ values below 0.5. The results of the TLC-DB assay with the DPPH radical suggest that, in addition to carotenoids, other unidentified compounds with antioxidant properties are present in the studied lipophilic fractions (Figure 5).

### 3.8. Quantitative Analysis by TLC

The TLC quantitative analysis considered the sum of the areas of all bands corresponding to carotenoids (yellow bands in visible light) present in the obtained TLC chromatograms. Carotenoid content was expressed as β-cryptoxanthin. Among the extracts analyzed, the highest carotenoid concentrations were recorded using the TLC method in cloudberry fruits, regardless of their place of origin and harvest (54.78–54.88 mg/100 g). A two-fold lower carotenoid content was confirmed in the *R. idaeus* species ‘Jantar’ (26.11 mg/100 g), ‘Husaria’ (21.29 mg/100 g), and ‘Poranna Rosa’ (19.21 mg/100 g), although this content was significantly higher than in the remaining fruits, especially black raspberry fruits (8.36–16.42 mg/100 g). The lowest carotenoid content was recorded for the *R. chingii* species (5.32 mg/100 g (Table 5)).

## 4. Discussion

In the literature, the importance of red raspberries (*Rubus idaeus*) and black raspberries (*Rubus occidentalis*) as rich sources of anthocyanins and ellagitannins with strong antioxidant potential has been repeatedly highlighted [34,35,36]. Much less attention has been paid to carotenoids, which—although widely present in many fruits and vegetables—have not yet been studied in detail in raspberries, even though they are also associated with antioxidant properties [37]. The few available reports indicate lutein and β-carotene as the predominant carotenoids in red raspberries. However, differences in carotenoid profiles among red raspberry cultivars have not been examined in detail. To date, the carotenoid composition of black raspberries remains unknown. The lack of studies on carotenoids in raspberries is partially related to methodological limitations, as carotenoids, being lipophilic compounds require specific extraction procedures and more advanced separation techniques than polyphenols, particularly under conditions that prevent oxidative degradation [12,38,39].

The analyses performed using the developed methods—namely HPLC-DAD-ESI-MS, off-line TLC/HPLC-DAD-ESI-MS, and TLC—revealed clear differences in the carotenoid composition, mainly xanthophylls, between the examined *Rubus* species and cultivars/hybrids. The combination of the off-line HPLC-DAD-ESI-MS method with semipreparative TLC enabled, for the first time, the identification of β-apo-10’-luteinal (6) as a component of most *R. idaeus* fruits analyzed. The exception were fruits of *R. occidentalis* cultivars, which did not contain this compound (Figure 3). Moreover, the combination of off-line TLC/HPLC-DAD-ESI-MS allowed in ‘Jantar’ cultivar fruits the detection of *trans*-lutein, as well as *cis*-lutein and lutein dicaprylate isomer (probable structure) (Table 4, Figure 2).

β-apo-10′-luteinal (6) belongs to the group of apo-carotenoids, specifically, it is classified as an apo-luteinoid/apo-zeaxanthinoid). Apo-zeaxanthinals/apoluteinals are colored, short-chain metabolites formed as a result of oxidative cleavage of the two terminal β or ε rings in the structure of lutein, and of the two terminal β rings in the symmetrical structure of zeaxanthin (the only structural differences between the two carotenoids) [31]. Therefore, unlike zeaxanthin, lutein can form either β- or ε-apo-luteinoids. Using the off-line TLC/HPLC-DAD-ESI-MS method, the presence of three apo-zeaxanthinals namely: β-apo-12’-zeaxanthinal (3), β-apo-10′-zeaxanthinal (4), and β-apo-8’-zeaxanthinal (5) (Table 4, Figure 3), was also demonstrated in the tested dietary supplement Strong Zeaxanthin. The tR value of β-apo-10′-zeaxanthinal (4) differs from that of β-apo-10′-luteinal (6), what’s associated with the fact that these compounds may originate from different lutein/zeaxanthin isomers. The multiple conjugated double bonds in the polyene chain of carotenoids make carotenoids susceptible to oxidative cleavage, leading to their degradation and the production of apocarotenoids [12,31,40]. Carotenoid degradation can occur through nonspecific mechanisms, such as photochemical oxidation, as well as oxidation by nonspecific enzymes, such as lipoxygenases and peroxidases. In addition, carotenoids can undergo specific enzymatic oxidative cleavage catalyzed by carotenoid dioxygenases (CCDs). The activity of specific CDDs results in, among others, β-10′-apocarotenoids produced by asymmetric enzymatic cleavage of the 9′,10′ double bond of the relevant carotenoids, e.g., β-carotene and, in the case of the studied raspberry fruits—lutein [40]. This is the first report of the occurrence of β-apo-10′-luteinal (6) in the carotenoid complex of raspberry fruit, but only in the fruits of both yellow- and red-fruited *R. idaeus* cultivars and in the fruits of two *R. occidentalis* and *R. idaeus* hybrids, which originate from the same source and breeding (producer ‘Niwa’, Brzezna, Poland). With reference to the enzymatic degradation process and the formation of β-apo-10′-luteinal as a result, it should be emphasized that β-ionone will also be produced [41]. In an attempt to explain the presence of β-apo-10′-luteinal as the dominant compound in the studied plant material, in addition to the above-mentioned processes of enzymatic or non-enzymatic degradation of the parent carotenoid lutein, the participation of other factors, such as environmental influences or additional genetic conditions, cannot be excluded [42]. The group of apo-luteinoids (apo-zeaxanthinoids) includes aldehyde compounds (apo-luteinals), acids (apo-luteinolic acids), and alcohols (apo-luteinols). Previous literature data report the natural occurrence of apo-luteinals and apo-luteinols in peppers, *Allophylus psilospermus*, rose petals, and avocados, among others [43,44]. A representative of the group of acids, β-apo-10′-luteinolic acid, is found in *Boronia megastigma* flowers [45]. Apo-carotenoids constitute a very broad and structurally diverse group, as they virtually encompass all cleavage products of different parent 40-carbon carotenoids, ranging from essential phytohormones such as abscisic acid (ABA) and strigolactones, to a variety of plant pigments, aromas (α- and β-ionones) and defense compounds [46,47]. For this reason, according to some authors, the term *apocarotenoids* is a traditional, non-systematic name, because it covers a range of different derivatives that arise as products of their enzymatic or non-enzymatic degradation [17,48]. It has been shown that apo-carotenoids can act as signaling molecules in plants, regulating processes related to oxidative stress and responses to environmental factors. Additionally, it has been suggested, that some of these compounds may exhibit biological activity in animal systems as well, acting as ligands for nuclear receptors (RAR—Retinoic Acid Receptors, RXR—Retinoid X Receptors) and modulating the expression of genes involved, among others, in cell proliferation and differentiation, as well as in anticancer effects [49]. For some apocarotenoids, a regulatory effect on lipid and cholesterol metabolism has been demonstrated. Apocarotenoids can modulate the expression of SREBP-1, LXRα, and PPARγ genes, influencing lipogenesis and fatty acid oxidation [49,50]. In animal models, reductions in triglyceride levels and improvements in lipid profiles have been observed. Furthermore, a positive effect on insulin resistance has also been reported. In mouse studies, β-apo-10′-carotenal reduced the expression of pro-inflammatory cytokines in adipocytes and improved insulin sensitivity [49].

The developed TLC and HPLC-DAD-ESI-MS methods for the analysis of xanthophylls can be considered as complementary. Their use enables the identification of cis/trans isomers of lutein, zeaxanthin, and selected apo-carotenoids [β-apo-10′-luteinal and β-apo-12′-zeaxanthinal (3), β-apo-10’-zeaxanthinal (4), and β-apo-8′-zeaxanthinal (5)] in raspberry fruits, initially against standards by TLC and further supported by UV and MS spectral data using HPLC-DAD-ESI-MS (Table 4, Figure 3). Previous reports indicated that conventional HPLC-DAD analysis does not always allow the separation of lutein and zeaxanthin in a way that allows the simultaneous identification of both compounds present in the matrix [51]. The developed HPLC separation conditions are typical for the RP technique, in terms of both a C-18 modified silica column and the chosen solvent mixture as the mobile phase. It was not necessary to use buffered mobile phases for the separation of standard carotenoids [52]. A limitation of the developed HPLC method was the preparation of samples in methanol, which does not allow the analysis of more lipophilic carotenes. On the other hand, the presence of individual carotenes (e.g., β-carotene) in complexes of these compounds with a simple composition can be assessed using the developed TLC chromatographic system. However, it should be emphasized, that carotenoid profiles are most often multicomponent and quantitatively diverse, which requires the use of more advanced techniques than TLC, including HPLC-MS/MS [53]. The application of off-line TLC/HPLC-DAD-ESI-MS enabled the unambiguous identification of trans-lutein, zeaxanthin, and β-apo-10′-luteinal and cis -lutein in some of the analyzed fruits (Figure 3, Table 4). The off-line TLC/HPLC method proved useful for identification of carotenoids, present in fruits at much significantly lower concentrations than β-apo-10′-luteinal or trans-lutein, but co-eluting with them in TLC chromatograms as single bands, namely *cis*-lutein and lutein dicaprylate. A similar approach to carotenoid analysis was previously suggested by Mercadante et al. in studies on xanthophyll esters in paprika fruits. In *R. idaeus* fruits (‘Husaria’, ‘Delniwa’, ‘Jantar’, ‘Promyk’, ‘Poranna Rosa’), the presence of β-apo-10′-luteinal was demonstrated for the first time; it was the dominant compound, accompanied by trans-lutein and cis-lutein occurring at low concentrations, particularly in yellow-fruited cultivars (‘Jantar’, ‘Poranna Rosa’, ‘Promyk’) (Figure 3, Table 4). In the two-component xanthophyll profiles of R. occidentalis fruits (‘Niwot’, ‘MacBlack’), either lutein or zeaxanthin predominated, depending on the cultivar. Moreover, the presence of β-apo-10′-luteinal was detected in interspecific hybrids *R. idaeus*/*R. occidentalis* R1314701 and *R. occidentalis*/*R. idaeus* R1613411, together with trans-lutein and zeaxanthin, with β-apo-10′-luteinal being the dominant compound in the fruits of the former hybrid [53]. The carotenoid profile of *R. idaeus* hybrids R1616002 and R1981802 was identical to that of *R. idaeus* fruits (Figure 3).

Against standards under the conditions of the developed TLC method, due to differences in the quantitative composition of carotenoid profiles, only zeaxanthin could be identified in *R. occidentalis* ‘Niwot’ fruits and *trans*-lutein in *R. occidentalis* ‘Heban’. In addition, the β-apo-10′-luteinal band was well separated from other carotenoid bands on TLC chromatograms of extracts from the *R. idaeus* cultivars ‘Jantar’, ‘Poranna Rosa’, ‘Promyk’, and the red-fruited cultivars ‘Husaria’ and ‘Delniwa’, as well as from the fruits of hybrids *R. idaeus* R1616002 and R1981802. At the same time, on TLC chromatograms the band corresponding to β-apo-10′-luteinal was present alongside the co-eluting *trans*-lutein and zeaxanthin band in the fruits of the hybrids *R. occidentalis*/*R. idaeus* ‘R1613411’ and *R. idaeus*/*R. occidentalis* ‘R1314701’. Using the β-carotene standard, its presence was confirmed in the analyzed cloudberry (*Rubus chamaemorus)* fruits (Figure 4).

β-apo-10′-luteinal belongs to the group of apo-carotenoids, which were initially considered to be products of oxidative degradation of lutein [42]. Raspberries are among the few fruits, in which an intensive increase in the level of apo-carotenoids is observed during ripening. The demonstrated variation in the presence of β-apo-10′-luteinal in the analyzed fruits may be related to the presence or absence of cyanidin-derived anthocyanins, which are strong natural antioxidants [25]. The results of chromatographic analyses of carotenoids in raspberry fruits may indicate the role of anthocyanins as antioxidants that protect these compounds from degradation processes. In black raspberries, characterized by a high concentration of these pigments, carotenoids remain more stable, which limits the formation of apoluteinals. In yellow-fruited cultivars, the absence of anthocyanins promotes increased accumulation of carotenoids and their oxidative degradation to apo-carotenoids. Similar relationships have been reported in studies on other berries, where the presence of anthocyanin pigments correlated with the stability of other secondary metabolites [54,55].

The carotenoid content determined both spectrophotometrically and by TLC in different raspberry fruits indicated that the concentration of these compounds decreases with increasing anthocyanin content. In *R. occidentalis* fruits, which contain the highest levels of anthocyanins [29], the carotenoid content was low and increased in red *R. idaeus* fruits, reaching the highest values in yellow-fruited cultivars. The highest carotenoid content, consistent with literature data [56], was observed in cloudberry fruits.

Considering the available literature data, most publications on the antioxidant properties of raspberries consider only polyphenolic compounds present in methanolic extracts [25,54]. There is a lack of studies evaluating the antioxidant potential of the lipophilic fraction of raspberries, which may partly contribute to the antioxidant activity of methanolic extracts as well as of the whole plant raw material. To date, only the antioxidant properties of a hexane extract from black raspberry seeds have been assessed, using DPPH, FRAP, and ABTS tests [57]. In our studies, a hexane–acetone mixture (1:1, *v*/*v*) was used—the addition of the hydrophobic component (hexane) was determined by the lipophilic nature of the extracted carotenoid compounds and by the need to exclude anthocyanins from the obtained extracts. The antioxidant activity of the hexane–acetone extracts from the analyzed raspberry fruits was evaluated using DPPH, ABTS, and FRAP assays. The DPPH test allows the determination of the antioxidant capacity of hydrophobic systems, while the FRAP test measures hydrophilic compounds—in contrast to the ABTS test, which assesses the antioxidant activity of both hydrophobic and hydrophilic compounds [57].

The antioxidant activity assays of lipophilic fractions from the analyzed raspberry fruits did not confirm the direct, dose-dependent effect of carotenoid levels on the observed activity [58,59,60]. Despite having the highest carotenoid content, cloudberry fruits did not show significantly higher activity in the FRAP or ABTS assays compared to red raspberry fruits, which in some cases contained nearly half the carotenoid levels, e.g., the cultivar ‘Husaria’. Comparable antioxidant activity in both FRAP and ABTS assays was also observed in yellow *R. idaeus* fruits, despite their lower carotenoid content, which was in some cases threefold lower, as in the cultivar ‘Promyk’. The antioxidant properties of lipophilic fractions from fruits of different *Rubus* species, their cultivars, and hybrids ranged as follows: in the ABTS assay, from higher (*R. occidentalis*/*R. idaeus* R1613411) to approximately to lowest (*R. idaeus* ‘Promyk’, ‘Husaria’) activity, and in the FRAP assay, from lowest (*R. chingii, R. occidentalis*/*R. idaeus* R1613412, *R. occidentalis* ‘Jewel’, ‘Niwot’, ‘MacBlack’) to highest (*R. idaeus* ‘Husaria’). No significant differences were observed among the lipophilic fractions from the analyzed fruits in the DPPH assay, ranging from approximately (od *R. chingii*, *R. occidentalis*/*R. idaeus* R1613411, *R. occidentalis* ‘Jewel’, ‘Bristol’, ‘MacBlack’—weakest activity to *R. idaeus* ‘Husaria’—twice as strong antioxidant activity). According to some authors, carotenoids do not exhibit DPPH radical-scavenging activity [9]. Other studies report that the DPPH radical-scavenging activity decreases in the following order: lycopene > β-cryptoxanthin > α-carotene > β-carotene > zeaxanthin > lutein [61]. The same authors emphasize that the assessment of DPPH radical-scavenging activity by carotenoids should be carried out at a wavelength of 580 nm, without using a carotenoid standard solution as a reference [62].

The observed differences in antioxidant activity, independent of carotenoid content, indicate the presence of compounds other than carotenoids and polyphenols (anthocyanins, ellagitannins, flavan-3-ols) that have not yet been identified in raspberry fruits, which exhibit both free radical-scavenging and radical-inhibiting effects. The performed TLC-DB assay using the DPPH radical further supports this conclusion.

## 5. Conclusions

The conducted study demonstrated that *Rubus* fruits constitute a valuable source of carotenoids with variable content depending on the species and cultivar/hybrid. The application of HPLC-DAD-ESI-MS and *off-line* TLC/HPLC methods enabled the identification of *trans*-/*cis*-lutein, zeaxanthin, β-carotene, and—for the first time in raspberries—β-apo-10’-luteinal. The results suggest that anthocyanins, as antioxidants, may exert a protective effect on carotenoids and limit their degradation to apo-carotenoids. The antioxidant activity of lipophilic fractions from the fruits, assessed by ABTS and FRAP assays, was variable, in contrast to the activity measured in the DPPH assay. TLC bioautography with the DPPH radical indicated that, in addition to carotenoids, other compounds with antioxidant activity may be present in the lipophilic fractions of raspberry fruits. Therefore, further studies on carotenoid compounds in lipophilic fractions of raspberry fruits should be conducted, including the successive stages of fruit ripening.

## Figures and Tables

**Table 1:** Characteristics of the examined fruits from various raspberry species, cultivars, and hybrids (parentage, fruit color, origin) [25].

Cultivar/Color of Fruit	Origin of the Cultivar	Pedigree * op—Free Dusting
*R. occidentalis* ‘Jewell’/black	Brzezna/Poland—from Niwa berry farm	‘Dundee’ × (Bristol × Dundee)’
*R. occidentalis* ‘Niwot’/black	Brzezna/Poland—from Niwa berry farm	A complex cross between two breeding clones from a natural environment of origin in the USA
*R. occidentalis* ‘Mac Black’/black	Brzezna/Poland—from Niwa berry farm	—
*R. occidentalis*/*R. idaeus* R1613411/Purple	Brzezna/Poland—from Niwa berry farm	‘Jewell’ × R121304 (‘Litacz’ (‘Bristol’ × op) × purple raspberry × op)
*R. occidentalis*/*R. idaeus* R1613412/purple	Brzezna/Poland—from Niwa berry farm	‘Jewel’ × R121304 (‘Litacz’ × purple raspberry) × op
*R. idaeus*/ *R. occidentalis* R1314701/purple	Brzezna/Poland—from Niwa berry farm	‘Litacz’ × ‘Sokolica’
*R. occidentalis* ‘Heban’ (R139501)/black	Brzezna/Poland—from Niwa berry farm	(purple raspberry × ‘Polka’) × op
*R. occidentalis* ‘Bristol’/black	Nałęczów/Poland Economic entity „Barbara Rusiecka-Górniak’	—
*R. idaeus* R1616002/red	Brzezna/Poland—from Niwa berry farm	R1634401 × ‘Polana’
*R. idaeus* ‘Husaria’/red	Brzezna/Poland—from Niwa berry farm	R120701 × ‘Sokolica’
*R. idaeus* ‘Delniwa’/red	Brzezna/Poland—from Niwa berry farm	‘Polka’ × R1211101
*R. idaeus* ‘Poranna rosa’/yellow	Brzezna/Poland—from Niwa berry farm	89112(83291 × ‘ORUS’ 10 98-1)
*R. idaeus* ‘Jantar’/yellow	Brzezna/Poland—from Niwa berry farm	R126107 (‘Heritage’ × ‘Polesie’)
*R. idaeus* ‘Promyk’/yellow	Brzezna/Poland—from Niwa berry farm	Poemat × R127302 (Pingvin × op)
*R. idaeus* R1981802/yellow	Brzezna/Poland—from Niwa berry farm	—
*R. chamaemorus*	Finland	—
*R. chamaemorus*	Sosnówka/Poland Lubeskie Zioła, Polska	—
*R. chingii*	China	—

* data provided by the breeder Niwa Berry Breeding Ltd. (Brzezna/Poland).

**Table 2:** Validation parameters for quantitative determination of β-cryptoxantin in fruits of *Rubus* species.

Parameter	β-cryptoxanthin
LOD [ng/point]	40
LOQ [ng/point]	140
Linearity [ng/point]	140–600
Interday precision (%CV)	4.82
Intraday precision (%CV)	7.20
R^2^	0.992–0.997
Calibration curve	y = 254.525x + 14311

**Table 3:** Antioxidant capacity determined by DPPH, FRAP, and ABTS assays, along with the carotenoid profiles of the analyzed fruits from red, yellow, and black raspberries, cloudberries, and *Rubus chingii.*

Species/Variety	DPPH (mmol TE/g DW)	ABTS (mmol TE/g DW)	FRAP (mmol TE/g DW)	Content of Carotenoids (%)
*R. occidentalis* ‘Bristol’ ^a^	0.066 ± 0.09 ^hijklmnop^	0.27 ± 0.06 ^jm^	0.26 ± 0.01 ^ghjklmop^	7.24 ± 0.06 ^fghjlmnop^
*R. occidentalis* ‘Jewel’ ^b^	0.06 ± 0.02 ^hijklmnop^	0.2 ± 0.03 ^hijlmop^	0.2 ± 0.03 ^ghjklmop^	12.31 ± 0.17 ^gopq^
*R. occidentalis* ‘MacBlack’ ^c^	0.072 ± 0.07 ^ijklmop^	0.24 ± 0.05 ^jm^	0.22 ± 0.07 ^ghjklmop^	11.59 ± 0.09 ^gopq^
*R. occidentalis* ‘Niwot’ ^d^	0.087 ± 0.011	0.27 ± 0.03 ^jm^	0.22 ± 0.04 ^ghjklmop^	11.93 ± 0.05 ^gopq^
*R. occidentalis* ‘Heban’ ^e^	0.087 ± 0.017	0.34 ± 0.08	0.32 ± 0.07 ^j^	9.96 ± 0.03 ^ghiop^
*R. occidentalis*/*R. idaeus* R1613412 ^f^	0.07 ± 0.01 ^ijklmop^	0.25 ± 0.07 ^jm^	0.2 ± 0.05 ^ghjklmop^	12.98 ± 0.21 ^gopq^
*R. occidentalis*/*R. idaeus* R1613411 ^g^	0.063 ± 0.007 ^hijklmnop^	0.17 ± 0.05 ^hijlmop^	0.52 ± 0.06 ^abcdjnq^	29.79 ± 0.04 ^abcdefikmnopq^
*R. idaeus*/*R. occidentalis* R1314701 ^h^	0.096 ± 0.009 ^abgq^	0.41 ± 0.11 ^bg^	0.52 ± 0.08 ^abcdjnq^	17.55 ± 0.06 ^aeikopq^
*R. idaeus* ‘Delniwa’ ^i^	0.115 ± 0.06 ^abcfgq^	0.41 ± 0.07 ^bg^	0.33 ± 0.05 ^j^	7.36 ± 0.08 ^fghjlmnop^
*R. idaeus* ‘Husaria’ ^j^	0.122 ± 0.07 ^abcfgq^	0.46 ± 0.09 ^abcdfgknq^	0.8 ± 0.11 ^abcdefghiklmnopq^	18.23 ± 0.06 ^aeikopq^
*R. idaeus* R1616002 ^k^	0.111 ± 0.11 ^abcfgq^	0.25 ± 0.06 ^jm^	0.51 ± 0.07 ^abcdjnq^	8.69 ± 0.03 ^fghjlmnop^
*R. idaeus* ‘Jantar’ ^l^	0.115 ± 0.05 ^abcfgq^	0.4 ± 0.03 ^bg^	0.47 ± 0.05 ^abcdjnq^	20.14 ± 0.09 ^aeikopq^
*R. idaeus* ‘Promyk’ ^m^	0.118 ± 0.12 ^abcfgq^	0.47 ± 0.11 ^abcdfgknq^	0.55 ± 0.07 ^abcdjnq^	14.54 ± 0.11 ^gopq^
*R. idaeus* ‘Poranna Rosa’ ^n^	0.095 ± 0.04 ^abgq^	0.24 ± 0.04 ^jm^	0.26 ± 0.03 ^ghjklmop^	16.19 ± 0.08 ^gopq^
*R. chamaemorus* Poland ^o^	0.119 ± 0.09 ^abcfgq^	0.41 ± 0.07 ^bg^	0.51 ± 0.04 ^abcdjnq^	46.01 ± 0.06 ^abcdefghijklmnq^
*R. chamaemorus* Finland ^p^	0.122 ± 0.16 ^abcfgq^	0.43 ± 0.04 ^ag^	0.45 ± 0.06 ^abcdjnq^	46.27 ± 0.08 ^abcdefghijklmnq^
*R. chingii* ^q^	0.063 ± 0.06 ^hijklmnop^	0.27 ± 0.02 ^jm^	0.2 ± 0.09 ^ghjklmop^	4.51 ± 0.06 ^abcdefghijklnopq^

Expressed as mean + SD (*n* = 3) (%) dry weight, superscript letters (a–q) within the same column identify groups of varieties showing statistically significant differences in content (*p* < 0.05), statistically significant differences are observed between varieties marked with the same letter.

**Table 4:** Characteristics of chromatographic parameters TLC, HPLC-DAD-ESI-MS: R_F_, t_R_ (min), UV λ_max_ (nm), and *m*/*z* values of molecular ions [M·]^+^, [M + H]^+^, and [M + H − 18]^+^ for reference substances and carotenoids identified in the analyzed *Rubus* fruits.

Compound	TLC	HPLC		
	R_F_	t_R_ (min)	λ_max_ (nm)	MS (*m*/*z*) [M + H]^+^ [M]^+·^ [M + H − 18]^+^
β-apo-12′-zeaxanthinal (3)	0.38	5.56	427	367^+^ [M + H]^+^
β-apo-10’-zeaxanthinal (4)	0.38	6.19	308 (sh), 446	393^+^ [M + H]^+^
β-apo-8’-zeaxanthinal (5)	0.38	8.74	337 (sh), 455	433^+^ [M + H]^+^
β-apo-10’-apoluteinal (6)	0.39	12.14	310 (sh), 424	393^+^ [M + H]^+^
*Cis*-lutein (7)	0.35	13.48	325, 421 sh, 444, 472	568^+^ [M]^+^
*Trans*-lutein (1)	0.35	13.56	267, 331, 422 (sh), 445, 473	568.5^+^ [M]^+·^ 551.5^+^ [M + H − 18]^+^
Zeaxanthin (2)	0.33	14.17	277, 340, 427 (sh), 451, 474	569.5^+^ [M + H]^+^ 568.5^+^ [M]^+·^
Isomer of lutein dicaprylate (8)	0.35	14.55	330, 417 (sh), 443, 470	677^+^ [M + H − 144]^+^ 676^+^
β-cryptoxanthin (9)	0.56	24.10	276, 340, 422 (sh), 448, 475	553.5^+^ [M + H]^+^ 552.5^+^ [M]^+·^
β-carotene (10)	0.74	39.35	280, 337, 424 (sh), 449, 477	535^+^ [M]^+·^

**Table 5:** Carotenoid content in the studied *Rubus* fruits determined by the TLC method with analysis of image (videoscanning).

Species/Variety	Total Carotenoid Content by TLC Method
*R. occidentalis* ‘Bristol’ ^a^	8.36 ± 0.11 ^bfghjlmnop^
*R. occidentalis* ‘Jewel’ ^b^	16.42 ± 0.22 ^akopq^
*R. occidentalis* ‘MacBlack’ ^c^	14.28 ± 0.12 ^gopq^
*R. occidentalis* ‘Niwot’ ^d^	12.99 ± 0.04 ^opq^
*R. occidentalis* ‘Heban’ ^e^	10.02 ± 0.05 ^op^
*R. occidentalis*/*R. idaeus* R1613412 ^f^	14.17 ± 0.08 ^gopq^
*Rubus occidentalis*/*Rubus idaeus* R1613411 ^g^	29.79 ± 0.04
*R. idaeus*/*R. occidentalis* R1314701 ^h^	18.21 ± 0.14 ^aopq^
*R. idaeus* ‘Delniwa’ ^i^	9.21 ± 0.11 ^bgjlmnop^
*R. idaeus* ‘Husaria’ ^j^	21.29 ± 0.1
*R. idaeus* R1616002 ^k^	9.54 ± 0.07 ^bgjlmnop^
*R. idaeus* ‘Jantar’ ^l^	26.11 ± 0.09
*R. idaeus* ‘Promyk’ ^m^	16.22 ± 0.11
*R. idaeus* ‘Poranna Rosa’ ^n^	19.21 ± 0.08 ^aopq^
*R. chamaemorus* Poland ^o^	54.78 ± 0.06 ^abcdefghijklmq^
*R. chamaemorus* Finland ^p^	54.88 ± 0.08 ^abcdefghijklmq^
*R. chingi* ^q^	5.32 ± 0.06 ^bcfghjlmnopq^

Expressed as mean + SD (n = 3) (%) dry weight, superscript letters (a–q) within the same column identify groups of varieties showing statistically significant differences in content (*p* < 0.05), statistically significant differences are observed between varieties marked with the same letter.

## Data Availability

The original contributions presented in this study are included in the article. Further inquiries can be directed to the corresponding author.
